# Standardizing Intestinal Permeability Assessment: Optimization of Gluten Dose and Urine Collection Times for u-GIP and Lactulose:Mannitol Ratio in Healthy Volunteers

**DOI:** 10.3390/ijms27052286

**Published:** 2026-02-28

**Authors:** Raquel Rodríguez-Ramírez, María Auxiliadora Fernández Peralbo, Ángel Cebolla, Carolina Sousa

**Affiliations:** 1 Biomedal S.L., 41900 Seville, Spain; raquel.rodriguez@biomedal.com (R.R.-R.); auxi.fernandez@biomedal.com (M.A.F.P.); angel.cebolla@biomedal.com (Á.C.); 2 Inorganic Chemistry Department, Faculty of Science, University of Granada, 18011 Granada, Spain; 3 Department of Microbiology and Parasitology, Faculty of Pharmacy, University of Seville, 41012 Seville, Spain

**Keywords:** gluten immunogenic peptides (GIPs), intestinal permeability, lactulose:mannitol ratio (LMR), gluten dose, urine collection protocols

## Abstract

Urinary gluten immunogenic peptides (u-GIPs) have been proposed as a complementary marker to classical intestinal permeability tests based on lactulose, mannitol, and the lactulose:mannitol ratio (LMR). However, the effects of gluten dose, urine collection interval, and sampling strategy on their performance remain insufficiently defined. This study evaluated these variables to support protocol standardization. Data from four standardized protocols including 46 healthy adults exposed to 0, 2, 4, or 10 g of gluten were analyzed. All participants ingested fixed doses of lactulose and mannitol. Urine was collected cumulatively (0–6 h and 0–15 h) or by individual voids. u-GIP levels were measured by lateral-flow immunoassay, and lactulose and mannitol by ion chromatography. u-GIP excretion showed a clear dose dependence. Lactulose excretion increased transiently only at the 10 g dose during the 0–6 h interval, while mannitol excretion and LMR were unaffected. The u-GIP excretion index showed linear proportionality at the 2 g and 4 g doses but exhibited saturation kinetics at the 10 g dose. The 4 g dose showed the lowest interindividual variability. Sampling strategies yielded equivalent results. A 4 g gluten challenge combined with a 6 h urine collection demonstrated effectiveness in healthy volunteers and may be suitable for clinical application. Further research involving larger cohorts of both healthy individuals and patients with intestinal hyperpermeability is required to validate this method.

## 1. Introduction

The intestinal barrier plays a critical role in maintaining gastrointestinal and systemic homeostasis by regulating the passage of nutrients, microorganisms, and macromolecules between the lumen and the internal milieu [1]. Disruption of this barrier, clinically defined as increased intestinal permeability (often colloquially termed “leaky gut” [2]), has been associated with multiple pathological conditions, including celiac disease, non-celiac gluten sensitivity, irritable bowel syndrome, chronic inflammatory disorders, and metabolic diseases [3,4]. Despite its clinical relevance, the lack of a “gold standard” for its assessment remains a significant hurdle in gastroenterology [5].

The lactulose–mannitol ratio (LMR) remains the most widely utilized non-invasive method for assessing intestinal permeability, based on the differential absorption and urinary elimination of two non-metabolizable sugars [6]. Nevertheless, despite its broad application, the LMR test is associated with several critical limitations: protocols are not universally standardized, accepted cut-off values are lacking, specialized analytical equipment is required, and the molecular sugars used have limited physiological relevance as immunoactive reference probes capable of inducing inflammatory responses in patients [5,7]. Furthermore, while current LMR protocols often stop at 5 or 6 h, the “tail” of lactulose excretion may carry vital information that short protocols miss, potentially explaining high inter-study variability. These issues contribute to variability across studies and hinder their broader clinical applicability [8,9,10].

**Figure 1:**
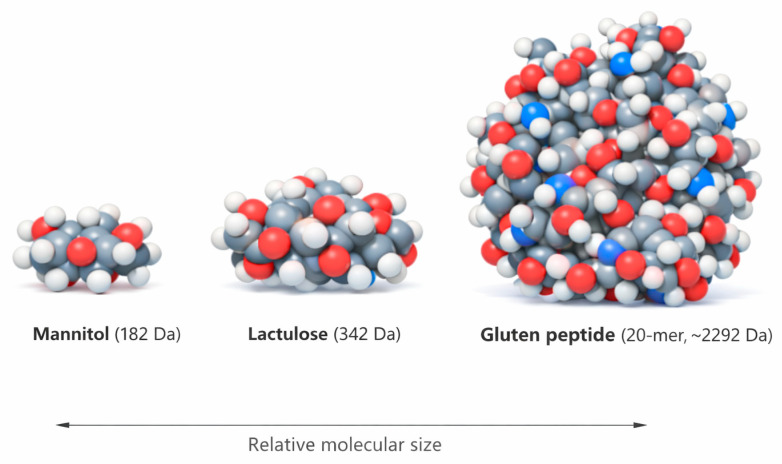
Relative molecular size of classical and gluten immunogenic intestinal permeability probes. Molecular size comparison between mannitol and lactulose, used in the lactulose–mannitol ratio (LMR) test, and a representative gluten immunogenic peptide (20-mer).

Recently, urine gluten immunogenic peptides (u-GIPs) have been proposed as a valuable tool for monitoring dietary gluten exposure [11] and may also provide insight into aspects of intestinal barrier function specifically related to food biomolecules. Previous work has demonstrated that GIPs can cross the intestinal barrier and be detected in urine following gluten ingestion, making them a physiologically relevant model of macromolecular translocation [12], particularly when compared with the much smaller probes used in the LMR test (Figure 1). Previous studies from our group reported comparable temporal dynamics between u-GIPs and lactulose and higher intraindividual reproducibility of u-GIPs compared with LMR [13,14].

Although significant progress has been made, several critical issues remain unresolved: (i) the impact of gluten dose on u-GIP excretion under controlled conditions, (ii) the optimal urine collection interval to reduce variability without compromising protocol implementation, and (iii) the assessment of the potential influence of gluten peptides on paracellular permeability during the procedure through simultaneous determination of LMR values. To address these questions, this study evaluates the impact of four gluten doses (0, 2, 4, and 10 g) and two collection windows (0–6 h and 0–15 h) on the excretion of u-GIPs and LMR.

## 2. Results

### 2.1. Baseline Urinary Profiles of u-GIP and Permeability Probes

We investigated the influence of gluten dosage on the assessment of intestinal permeability with the aim of establishing reference values for a presumed healthy population, minimizing variability as much as possible. A total of 182 urine samples were collected from all participants. Excluding baseline urination, the average number of urinations per participant during the 15 h period in the first study was determined to be 8 ± 3. Importantly, baseline urine samples showed non-detectable levels of u-GIPs and lactulose, providing analytical confirmation of successful adherence to the pre-study dietary requirements. However, an average baseline of 3.36 ± 5.21 mg of mannitol was detected in these samples, highlighting the challenges of adhering to a mannitol-free diet despite recommendations to exclude foods rich in mannitol across the two studies. This persistent baseline mannitol underscores the inherent limitations of this sugar as a marker compared to the cleaner baseline offered by u-GIPs.

### 2.2. Impact of Gluten Dose and Sampling Periods on Intestinal Permeability Markers and u-GIP Excretion

**Figure 2:**
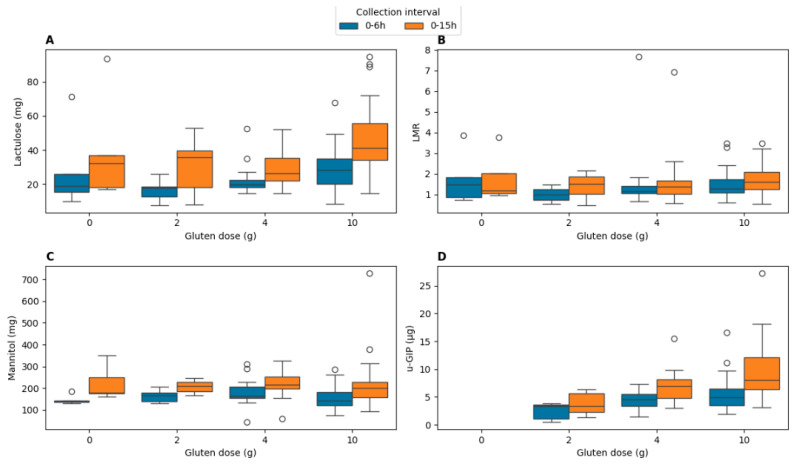
Comparative urinary excretion of intestinal permeability markers and u-GIP across gluten doses and collection intervals. (**A**) Lactulose (mg); (**B**) lactulose:mannitol ratio (LMR); (**C**) mannitol (mg); and (**D**) gluten immunogenic peptides (GIP, µg). Data represent the 0–6 h (early phase) and 0–15 h (extended phase) intervals following ingestion of 0 g, 2 g, 4 g, and 10 g of gluten. Box plots display medians, interquartile ranges (IQRs), and individual outliers (dots) identified by the 1.5× IQR method. Figure 3 provides the formal statistical validation (*p*-values) for the quantitative differences visualized in these box plots. Note the robust dose-response observed for u-GIPs and the discriminatory stability of the LMR across both timeframes.

The urinary excretion of lactulose, mannitol, the LMR and GIP was evaluated to establish the sensitivity of each marker according to the administered gluten dose (0 g, 2 g, 4 g, 10 g) and the urine collection interval (0–6 h and 0–15 h). The results are displayed in Figure 2.

Outliers were identified using the interquartile range (IQR) method and are shown as individual points in Figure 2. Some outliers were driven by isolated cases with disproportionately low mannitol relative to lactulose, resulting in elevated LMR values.

For clarity and to facilitate quantitative comparison, median and interquartile range (IQR) values for urinary GIP and lactulose excretion across gluten doses and collection intervals are additionally summarized in Table 1.

**Figure 3:**
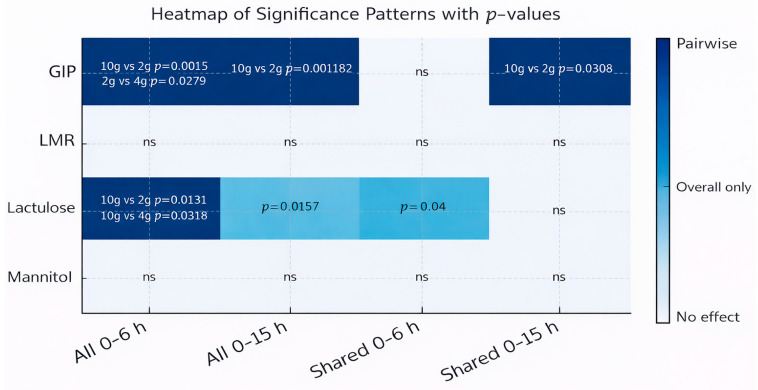
Heatmap synthesizing the significance patterns observed across varying gluten doses, time intervals, and participant cohorts. Darker blue cells indicate significant pairwise differences between specific gluten doses, lighter blue indicates significance at the global test level (ANOVA/Kruskal–Wallis) without specific pairwise contrasts, and white cells indicate the absence of a statistically significant effect. Cell labels display precise *p*-values for significant pairwise comparisons, as well as for overall effects in cases where non-pairwise differences are observed. Statistical tests were tailored to data distribution: one-way ANOVA with Tukey’s post hoc test for normally distributed variables and Kruskal–Wallis test with Dunn’s pairwise comparisons for non-normal variables. “All participants” refers to the full study cohort (*n* = 46). “Shared participants” denotes the subset of individuals who completed all dosing conditions (*n* = 4). Abbreviations: GIP, gluten immunogenic peptide; LMR, lactulose:mannitol ratio.

To further integrate these findings and formally assess dose-related effects across cohorts, a comparative significance analysis was performed (Figure 3). The impact of gluten dose on the excretion of u-GIPs, lactulose, and mannitol and the LMR was evaluated across all participants and in the subset of four individuals who completed all experimental conditions (“Shared”). For u-GIPs, significant dose-related effects were observed in both the 0–6 h and 0–15 h intervals in the full cohort. In the shared participant subset, u-GIP showed a significant difference only at 0–15 h; however, this intraindividual result should be interpreted as exploratory due to the limited sample size. Overall, the 15 h period captures the complete pharmacokinetic tail, whereas the 6 h interval provides a representative measure when cohorts are sufficiently powered.

Lactulose exhibited significant gluten dose-related effects during the 0–6 h interval. No pairwise differences emerged for lactulose in the 0–15 h interval. Mannitol and LMR showed no significant effects regardless of the gluten dose in any interval or participant subset, consistent with the absence of highlighted cells in the heatmap.

These results indicate that the magnitude of gluten primarily modulates u-GIP recovery and, to a lesser extent, induces a transient increase in early-phase lactulose excretion, while the core markers of intestinal barrier integrity, mannitol and LMR, remain statistically invariant under the tested conditions.

### 2.3. Correlation Between Gluten Dose and Excretion Parameters

To determine whether increasing gluten intake leads to proportional changes in intestinal permeability markers, we evaluated the presence of a dose–response relationship between gluten dose and the urinary excretion of GIP, lactulose, mannitol, and the LMR. This analysis aimed to establish the linearity of the physiological response to gluten challenges under standardized conditions.

Spearman rank correlations between gluten dose and the excretion of u-GIP, lactulose, mannitol, and LMR are presented in Table 2. Correlations are shown both for all participants across the four studies to provide population-level insights and for the subset of four volunteers who completed all dosing sessions (“4 shared volunteers”) to evaluate intra-individual consistency.

Across the whole cohort, u-GIP excretion showed a weak positive correlation with gluten intake at 0–6 h, which progressed to a moderate positive correlation at 0–15 h. This progression indicates that while u-GIP is sensitive to dose in the acute phase, its quantitative proportionality is significantly enhanced when the full excretion window is captured. Notably, lactulose exhibited moderate correlations with gluten dose at both 0–6 h and 0–15 h. Mannitol showed no significant correlation in either interval, confirming that its absorption kinetics are independent of the co-administered gluten load. LMR correlations with gluten dose were weak, reaching statistical significance only at 0–6 h but not at 0–15 h.

In the subset of four shared volunteers, correlations were generally stronger but should be considered exploratory due to the limited sample size and inter-individual heterogeneity.

### 2.4. Correlation Analysis Between u-GIP and Established Intestinal Permeability Markers

Spearman’s correlation analyses were performed to explore the relationship between u-GIP excretion and classical intestinal permeability markers (LMR and lactulose) across both urine collection intervals (0–6 h and 0–15 h) and for each gluten dose. This cross-biomarker evaluation aimed to determine whether gluten-induced absorption kinetics of immunogenic peptides align with the paracellular transport of sugar markers.

At the 2 g dose, a strong positive correlation was observed between u-GIPs and LMR (ρ = 0.786, *p* = 0.036) during the 0–6 h interval. This relationship was not maintained in the extended 0–15 h interval and must be interpreted as exploratory due to the limited sample size (*n* = 7). A similar trend was observed between u-GIPs and lactulose (ρ = 0.702, *p* = 0.081) at this dose and interval.

For the higher antigenic loads (4 g and 10 g), no significant correlations were observed between u-GIPs and any permeability marker in either collection window.

### 2.5. Protocol Optimization and Methodological Standardization

#### 2.5.1. Comparative Analysis of Interindividual and Interstudy Variability

To identify the conditions that minimize variability in excretion measurements, interindividual and interstudy coefficients of variation (CVs) were calculated for u-GIP and LMR. As summarized in Table 3, the 4 g gluten challenge consistently yielded the lowest CVs in both the 0–6 h and 0–15 h collection intervals. Specifically, u-GIPs exhibited CVs of 34.86% and 34.43%, while LMR showed CVs of 25.37% and 26.49% for the 0–6 h and 0–15 h intervals, respectively. These data demonstrate that a 4 g dose significantly reduces biological and procedural variance compared to the 2 g and 10 g doses, providing the highest degree of precision for standardized clinical monitoring protocols.

#### 2.5.2. Validation of Urine Collection Strategies

The impact of urine sampling methodology on analyte recovery was evaluated by comparing individual sampling (each void collected separately and subsequently pooled) with direct pooled sampling (urine collected directly into a single container per interval) after ingestion of 4 g of gluten. Normality testing indicated that lactulose and u-GIP data were normally distributed under both collection methods, whereas mannitol showed a non-normal distribution in the individual collection group. Consequently, paired t-tests were applied for lactulose and u-GIP, and a Mann–Whitney U test was used for mannitol. No statistically significant differences were observed between the two collection strategies for any analyte (lactulose: *p* = 0.1681; mannitol: *p* = 0.0878; u-GIP: *p* = 0.1180). This lack of systematic bias confirms that both collection methods are interchangeable, allowing for greater flexibility in clinical settings without compromising data integrity.

These findings support the use of a 4 g gluten dose and either urine collection strategy for reliable assessment of u-GIP and LMR, providing a foundation for protocol standardization in clinical studies.

#### 2.5.3. The Gluten Immunogenic Peptides Leakage Index (GALI) as a Reference of Gut Permeability to Dietary Antigens

We aimed to obtain a normalized physiological constant with minimized variability to represent the reference range of normality of a healthy gut concerning the permeabilization to gluten antigenic peptides. To determine the most appropriate gluten dose for establishing a gut permeability referent value, we evaluated the Gluten Antigenic Leakage Index (GALI) across doses and collection intervals (0–6 h and 0–15 h). For descriptive analysis, we calculated the mean GALI for each dose–interval combination across participants (with at least 7 different volunteers per dose), along with the corresponding standard deviation.

As detailed in Table 4, GALI values exhibited a non-linear relationship with the administered dose. Mean GALI values for the 10 g dose were the lowest among all tested doses in both intervals: during the 0–6 h period, mean GALI levels were 54% and 58% lower compared to the 4 g and 2 g gluten doses, respectively; in the 0–15 h urine collection period, the reductions were 44% and 50% against the same respective doses (derived from the mean values reported in Table 4). The 2 g and 4 g gluten doses yielded similar mean GALI values in the initial 0–6 h period, while the extended collection interval showed that the 4 g dose reached approximately 90% of the value observed with the 2 g dose. Variability differed across dosing conditions, with the 2 g dose exhibiting the greatest dispersion and the 4 g dose demonstrating notably lower SD. We determined that the 4 g gluten dose provided the most reliable GALI values. Compared with the 2 g dose, it showed lower variability and greater statistical reliability due to a larger sample size (*n* = 13), and it provided a stronger antigenic signal than the 10 g dose, making it the optimal choice for practical application.

## 3. Discussion

This study provides a comprehensive evaluation of how gluten dose, urine collection interval, and analytical strategy influence the urinary recovery of gluten immunogenic peptides (u-GIPs) and classical intestinal permeability markers. By integrating data from four standardized protocols, we identified the experimental conditions that offer the most consistent and physiologically meaningful measurements. Pooling data from multiple protocols may introduce some degree of heterogeneity due to minor differences in compound administration and analytical methods. Residual protocol-related variability cannot be fully excluded and should be considered a limitation.

The strict dietary control and standardized urine collection protocol enabled comparable results across the four studies. The absence of u-GIP and lactulose at baseline confirms adherence to the gluten-free diet, whereas the persistent detection of mannitol in all baseline urines, despite dietary restrictions, highlights the intrinsic limitation of this probe due to its ubiquitous presence in commonly consumed foods [15]. Although baseline mannitol levels were low relative to post-challenge excretion and are unlikely to materially affect LMR estimates, they contrast with the consistently negative u-GIP baseline. Consistent with previous reports, differences in dietary preparation and urine collection procedures can introduce methodological variability in LMR testing, underscoring the need for standardization [8,16,17,18].

The 0–15 h interval is crucial for a complete pharmacokinetic profile, especially in research settings, where it captures the full elimination kinetics and cumulative systemic exposure [12,19,20]. However, our results indicate that the 0–6 h interval provides a representative snapshot of the acute epithelial response, consistent with the physiological transit time through the proximal small intestine [21,22]. From a translational perspective, a 6 h collection may be more compatible with clinical feasibility [23]. Nevertheless, the clinical applicability of both collection intervals should be evaluated and validated in patient populations.

Beyond the influence of collection interval, gluten dose also shaped the excretion patterns of the evaluated analytes. The effect of gluten dose was reflected primarily in u-GIP excretion and, to a lesser extent, in lactulose, whereas mannitol and the LMR remained stable. This pattern is consistent with previous studies showing that urinary GIP excretion increases proportionally with the amount of gluten ingested [12]. The highest gluten dose (10 g) may selectively affect the absorption and elimination of lactulose. Transient gluten-induced modulations of permeability [24,25] cannot be ruled out as an explanation for the increased lactulose excretion [10]. For lactulose, dose-dependent differences were evident only in the early 0–6 h interval, consistent with reports describing short-term epithelial responses to gluten exposure [26,27,28]. However, these differences disappeared when extending the collection to the 0–15 h interval, indicating that the effect was time-limited. The stability of mannitol and the LMR across doses further supports the absence of sustained or global changes in intestinal permeability induced by gluten under the tested conditions. Notably, although lactulose excretion increased significantly at the 10 g dose, the LMR remained stable, suggesting that the magnitude of the rise was insufficient to overcome the physiological variance in mannitol excretion, which acts as the denominator in the ratio. Overall, these findings suggest that dose-related effects are more effectively quantified through u-GIP excretion kinetics, which specifically reflect the epithelial translocation and subsequent systemic availability of immunologically active peptides. In contrast, the stability of the LMR indicates preserved structural integrity of the paracellular pathway across the tested gluten range.

The positive correlation observed between u-GIP and the LMR at the lower gluten doses and within the early 0–6 h interval, although based on a limited number of observations, should be interpreted as exploratory and suggests that under conditions of low gluten exposure and during the initial duodenal absorption period, the best correlation between gluten immunogenic peptide passage and paracellular permeability markers occurred.

In healthy individuals, u-GIP variability was mainly driven by gluten intake, while global permeability remained stable, as reflected by moderate GALI variation. The 4 g gluten dose consistently showed lower coefficients of variation than the 2 g dose, indicating a ‘stability plateau’ that minimizes interindividual biological noise. At the highest gluten dose (10 g), GALI exhibited non-linear behavior, with reduced antigen leakage per gram of gluten, consistent with a saturation effect rather than a proportional dose–response. A similar capacity-limited handling of dietary substrates by the small intestine has been described for other nutrients (e.g., fructose), where high loads can saturate intestinal processing and alter downstream disposition [29]. Overall, the 4 g dose provided the most reliable balance between signal magnitude and variability, supporting its selection for methodological standardization.

Although this study does not include GALI data for a cohort of patients, we hypothesize that notable differences in GALI may exist among individuals with intestinal hyperpermeability. In a clinical trial with celiac patients following a gluten-free diet (2–4 g of gluten/day for 10 months), those patients who developed severe villus atrophy (villus height: crypt depth <1) had about 4 times more gluten peptide concentration of u-GIPs than volunteers with healthy intestine or celiac patients on a gluten-free diet [30]. This supports the potential of GALI as a normalized metric to assess the “leakiness” of the gut independently of total gluten intake.

Based on these findings, the second study was designed using the 4 g dose together with a simplified two-interval collection strategy (0–6 h and 6–15 h), improving feasibility by reducing sample handling. Both collection approaches, individual and pooled, provided equivalent estimates for all analytes. Overall, the combination of a 4 g gluten provocation with a stratified collection window, centered on the first 6 h, offers a robust and reproducible framework for evaluating u-GIP translocation, supporting future clinical evaluation pending validation in patient populations, while the optional extension to 15 h ensures maximum pharmacokinetic resolution for research application.

Given the commercial involvement of some authors, it should be noted that the conclusions of this work are supported by internally consistent dose- and time-dependent trends observed across multiple protocols and by parallel evaluation with established intestinal permeability markers. Further independent analytical validation and benchmarking against orthogonal methods will be important to strengthen confidence and support broader clinical translation. The 4 g protocol is currently being evaluated in an ongoing clinical study including patients with active celiac disease and healthy volunteers, with duodenal biopsies performed to assess villus atrophy. These results will inform the clinical applicability of the method.

## 4. Materials and Methods

### 4.1. Study Design

This work combines data from previously published studies [13,14] and two newly conducted experimental protocols, all designed to evaluate the urinary excretion of ingested markers under controlled dietary conditions. This integrative approach allowed for a systematic evaluation of how varying gluten loads and collection durations affect biomarker recovery. Data were pooled to increase statistical power across gluten doses and collection intervals.

All study protocols followed a standardized design consisting of two main phases: a washout period and an intake/collection phase. During the washout phase, participants adhered to a gluten-free diet for 32 h, including a final 8 h fasting period. They were also instructed to avoid dairy products and foods high in sorbitol and/or mannitol to minimize baseline interference in the LMR test. Compliance was assessed through food recall questionnaires, and urine samples collected prior to compound ingestion (T0) were analyzed to confirm the absence of the target analytes and establish a clean baseline for each participant.

Following the fasting period, participants ingested a solution containing lactulose (10 g), mannitol (1 g) and varying doses of gluten (0, 2, 4, or 10 g). The 10 g dose served as the primary reference based on previous data, while the lower doses (0, 2, and 4 g) were implemented in the new protocols to define the dose–response relationship and identify the optimal threshold for gluten antigen leakage. In one of the new studies, the same experimental design was repeated three times by each participant with decreasing gluten doses (0, 2 and 4 g), while maintaining constant amounts of lactulose and mannitol. In this case, urine was collected separately for each void throughout the 15 h post-ingestion period, as was also done in the previously published studies. In the second new study, participants followed the same protocol but ingested 4 g of gluten and urine was collected in two composite fractions corresponding to the 0–6 h and 6–15 h intervals.

**Figure 4:**
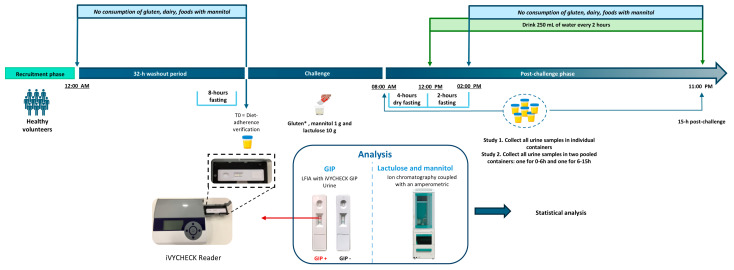
Study timeline illustrating the periods of fasting, ingestion of gluten at different doses (0, 2, 4, and 10 g), lactulose/mannitol intake, liquid consumption, and sample collection. * Gluten doses varied depending on the study condition.

After compound ingestion, participants remained fasted for 4 h and then followed a scheduled fluid intake regimen (250 mL every 2 h) to ensure adequate urine output for kinetic analysis. At the 6 h mark, they began a restricted diet (gluten-free, low in mannitol), which continued until the end of the collection period at 15 h post-dose. Dietary compliance and fluid intake were monitored throughout the study using structured dietary records. A schematic timeline of the protocol is shown in Figure 4.

### 4.2. Study Population

**Figure 5:**
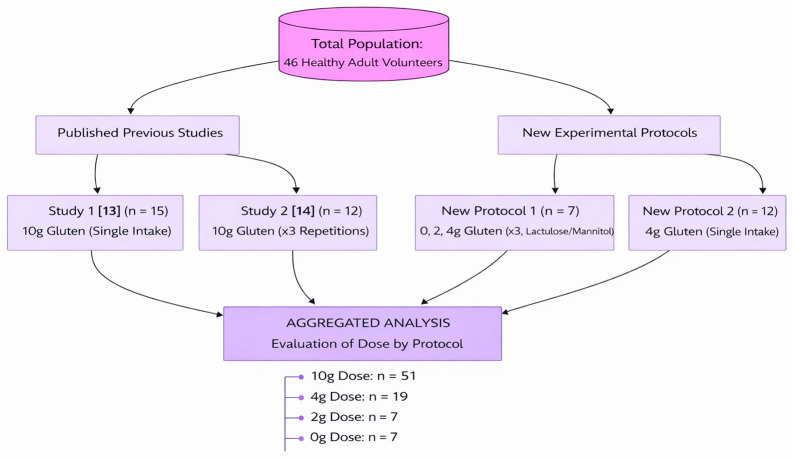
Distribution of participants across study protocols and aggregated analysis by gluten dose [13,14].

A total of 46 healthy adult volunteers participated across four study protocols. The cohort distribution was as follows: in the first previously published study [13], 15 individuals completed the protocol with a single intake of 10 g of gluten; in the second previously published study [14], 12 individuals repeated the same protocol three times, each with a 10 g gluten intake. Among the newly conducted studies, one included 7 participants who completed the protocol on three separate occasions, each time with a different gluten dose (0, 2 and 4 g), while maintaining constant lactulose and mannitol intake. The other new study involved 12 individuals who followed the protocol once with a 4 g gluten dose. Although the sample sizes varied across cohorts, the use of repeated-measures designs in the groups increased the statistical power for detecting dose-dependent changes. To further support analysis at higher exposure levels, data were aggregated from multiple clinical cohorts. The total number of protocol evaluations varied across doses, with the 10 g dose tested in two studies (*n* = 51), the 4 g dose in two studies (*n* = 19), and the 2 g and 0 g doses in a single study (*n* = 7 each). This distribution provides a high-powered analysis for the detection of gluten-induced changes at clinical doses (4–10 g). The distribution of participants across previously published and newly conducted protocols, as well as the aggregation of evaluations by gluten dose, is summarized in Figure 5. For clarity, a structured overview of the four study protocols, including dose allocation, repetition scheme, and urine collection procedures, is provided in Table 5.

Participants were included based on the following criteria: (1) age > 18 years; (2) absence of celiac disease (CD), nonceliac gluten sensitivity, food allergies, food intolerances, gastrointestinal diseases, metabolic disorders, cardiovascular diseases, or other systemic conditions that could affect intestinal permeability; (3) willingness to adhere to a strict diet regimen; and (4) ability to collect daily urine samples. To rule out CD and gluten-related disorders, all participants completed a symptom-based questionnaire [31] and underwent a CeliacDetect^®^ rapid test (Biomedal S.L., Seville, Spain) to ensure a seronegative baseline for anti-tTG IgA antibodies.

Exclusion criteria included (1) presence of concurrent pathologies.

This study was conducted in accordance with the guidelines of the Declaration of Helsinki. All participants provided written informed consent, and the study was approved by the local ethics committee (no. 1308-N-23).

### 4.3. Administration of Compounds

The compounds consisted of varying amounts of gluten powder (standardized organic wheat gluten, Biorganic, Toledo, Spain) and 1 g of mannitol (Acofarma, Madrid, Spain), both aliquoted in the laboratory, together with lactulose (Duphalac™, Abbott Laboratories, S.A., Madrid, Spain). The use of a single source of gluten powder ensured consistency in the GIP load across all participants. The ingestion procedure was as follows: 50 mL of water was added to the lactulose and mannitol bottles, shaken, and consumed entirely. Finally, 160 mL of natural orange juice was added to the gluten bottle to improve palatability and ensure total consumption of the dose; the mixture was then shaken and consumed completely.

### 4.4. Urine Collection

Rigorous instructions and materials were provided to ensure total urine recovery, which is critical for calculating absolute excretion amounts. The subjects were equipped with all necessary materials for urine collection, including plastic screw-capped containers, labels, cool bags, isothermal boxes and cool packs. The participants were instructed to collect the entire urine sample from each micturition, noting the date and time of collection. All urine samples were preserved in isothermal boxes with cool packs at 4–8 °C and deposited within 48 h of collection. Urinary GIP levels were confirmed to remain stable for up to 78 h when stored at 4–8 °C, based on testing of negative, borderline and positive samples across multiple time points. The samples were then frozen at −20 °C until processing. The GIP concentration in the urine remained stable throughout the freeze-thaw process. To maintain analytical integrity, all samples from the same participant were processed in the same batch to eliminate inter-assay variability.

Two different urine collection procedures were used depending on the study protocol. In the first, used in the two previously published studies and in one of the newly conducted protocols (gluten doses of 0, 2 and 4 g), the participants collected the total volume of each individual void over the 15 h post-ingestion period, recording the date and time of each micturition. In the second method, applied in the other new study (4 g gluten), the participants were instructed to collect all urine in two separate 24 h urine containers: one for the 0–6 h interval and the other for the 6–15 h interval.

### 4.5. Urine Analysis

The volume of each urine sample was recorded. When multiple containers were required for the same urination, the samples were mixed and homogenized. Additionally, some mixed samples were analyzed at 0–6 h and 0–15 h intervals. To prepare these mixtures, 10% of the volume from each container was used. In the study using 24 h containers, the total volume of each of the two collection intervals (0–6 h and 0–15 h) was recorded. Additionally, for this study, a pooled sample representing the full 0–15 h interval was prepared by mixing 10% of the volume from each of the two time-point containers.

To prevent bacterial growth, 100 µL of 1% chlorhexidine diacetate was added. Aliquots of 1 mL were stored at −20 °C until analysis. The concentration of GIPs in urine remained stable under these storage conditions and after freeze–thaw cycles.

#### 4.5.1. u-GIP Analysis

Qualitative analysis of GIPs in urine was conducted using a LFIA with iVYCHECK GIP Urine (Biomedal S.L., Seville, Spain), following the manufacturer’s guidelines. Thawed urine samples were homogenized and mixed with a conditioning solution. Subsequently, 100 µL of the mixture was added to the immunochromatographic cassette, and visual interpretation of results occurred after 30 min. A positive outcome was determined if the test line exhibited a red color accompanied by a green color on the control line. A negative result was confirmed when only the control line displayed a green color. The limit of detection determined by visual inspection was 2.50 ng GIP/mL urine. GIP concentration in urine was also assessed on the immunochromatographic strips using the iVYCHECK Reader (Biomedal S.L., Seville, Spain). Reader calibration was performed prior to urine analysis using α-gliadin 33-mer peptide as a standard. The dynamic range established for this method was 3.12–25 ng GIP/mL urine. The results were expressed in “ng GIP per mL of urine”. Each mixed sample (0–6 h, 6–15 h and 0–15 h intervals) was subjected to duplicate experiments. The results presented in this paper are expressed in absolute amounts, calculated by considering both the concentration and volume of each urine sample.

#### 4.5.2. The Gluten Antigen Leakage Index (GALI)

The gluten antigen leakage index (GALI) was defined as urinary u-GIP excretion (µg) normalized by the administered gluten dose (g), i.e., GALI (µg/g) = u-GIP excreted (µg)/gluten intake (g).

#### 4.5.3. Lactulose/Mannitol Analysis

Lactulose and mannitol analytes were determined using ion chromatography coupled with an amperometric detector, with a linear range of 10–1500 mg/L. The ion chromatograph system used a 930 Compact IC Flex (Metrohm, Herisau, Switzerland) equipped with a Metrosep Carb 2 column (5 µm, 4 × 150 mm) (Metrohm, Herisau, Switzerland). Chromatographic separation was performed using a mobile phase of 300 mM NaOH and 1 mM sodium acetate, operating in isocratic mode with a constant flow rate of 0.5 mL/min. The injection volume was 5 µL, the oven temperature was maintained at 30 °C, whereas the column compartment was set to 6 °C.

Detection was carried out using an amperometric detector operating at a temperature of 35 °C. The electrochemical cell was equipped with a Pd reference electrode and a gold working electrode.

The aliquots were thawed and agitated for one minute using a vortex mixer. Subsequently, they were centrifuged for 5 min at 5000 *g* to remove sediments. Fifty microliters of the supernatant were collected and brought to a final volume of 1 mL with water. The final dilution ratio was 1:20 (*v*/*v*).

The results presented herein are expressed as absolute amounts, calculated by considering both the concentration and the volume of each urine sample.

### 4.6. Statistical Analysis

Quantitative variable results were presented using both the mean (SD). The LMR values were then multiplied by 100.

Data distribution and variance homogeneity were assessed using the Shapiro-Wilk and Levene tests, respectively. Normally distributed data with equal variances were analyzed using one-way ANOVA, followed by Tukey’s post hoc test for pairwise comparisons. Non-normal data or data with unequal variances were analyzed using the Kruskal-Wallis test, with Dunn’s post hoc test for pairwise comparisons. Correlations were evaluated using Spearman’s rank correlation coefficient. All analyses were performed in Python (version 3.12; Python Software Foundation, Wilmington, DE, USA) using SciPy (version 1.16.3; SciPy community), statsmodels (version 0.14.6; statsmodels developers), and scikit-posthocs (version 0.12.0; scikit-posthocs developers). Statistical significance was set at *p* < 0.05.

## 5. Conclusions

This study evaluated both the optimal gluten dose and the appropriate urine collection intervals needed to develop a method for assessing intestinal paracellular permeability using gluten as a physiologically relevant immunoactive probe. Across four standardized protocols, a gluten dose of 4 g consistently produced the lowest interindividual and interstudy variability in healthy volunteers, making it the most reliable condition for comparative analysis under the tested conditions. Regarding collection timing, while the 0–15 h window provides a complete kinetic profile of most of the detectable urinary gluten peptides, the 0–6 h interval provided significant consistency in the results to consider its possible use as a practical protocol. This shorter window represents a pragmatic, potentially clinically viable strategy that balances analytical sensitivity with operational feasibility. Importantly, variations in the gluten dose did not affect the LMR, indicating that overall intestinal permeability was not substantially affected by gluten intake in healthy individuals.

The ability to detect gluten peptides using a lateral flow immunoassay and a reader renders this method more suitable for a point-of-care setting compared to the LMR test, which requires complex chromatography equipment. Clinical performance and diagnostic thresholds must be established in patient cohorts.

## Figures and Tables

**Table 1:** Excretion of GIPs and lactulose by dose and time: median and interquartile range (IQR).

Interval	Gluten Dose	u-GIPs (µg)	Lactulose (mg)
0–6 h	10 g	4.89 (3.54–6.45)	28.21 (20.13–34.78)
	4 g	4.58 (3.35–5.50)	19.91 (18.31–22.64)
	2 g	3.37 (1.07–3.66)	17.82 (12.69–18.42)
	0 g	NA	19.07 (15.61–25.89)
0–15 h	10 g	8.07 (6.37–12.16)	41.12 (34.29–55.79)
	4 g	7.02 (4.83–8.14)	26.36 (22.12–35.27)
	2 g	3.33 (2.33–5.68)	35.81 (18.16–39.47)
	0 g	NA	32.42 (18.29–36.89)

Median (IQR) of u-GIP and lactulose excretion by dose and time interval. u-GIP values are presented in micrograms (µg) and lactulose in milligrams (mg) for two collection intervals: 0–6 h and 0–15 h. “Not observed” for u-GIPs at 0 g indicates no excretion data for that dose. Abbreviations: u-GIPs, urinary gluten immunogenic peptides, NA: not applicable (no gluten intake; u-GIP not expected).

**Table 2:** Spearman correlation between gluten dose and excretion parameters, with corresponding *p*-values.

Parameter	Interval	Correlation * (All Volunteers)	Correlation * (4 Shared Volunteers)
GIP	0–6 h	Weak (ρ = 0.286; *p* = 0.016)	3/4 significant (ρ = 0.538–0.926; *p* < 0.05)
	0–15 h	Moderate (ρ = 0.484; *p* < 0.001)	3/4 significant (ρ = 0.845; *p* < 0.05)
LMR	0–6 h	Weak (ρ = 0.251; *p* = 0.031)	Non-significant (*p* > 0.05)
	0–15 h	Weak (ρ = 0.239; *p* = 0.057)	Volunteer 2 (ρ = 0.768; *p* = 0.044)
Lactulose	0–6 h	Moderate (ρ = 0.471; *p* < 0.001)	Volunteer 2 (ρ = 0.907; *p* = 0.002)
	0–15 h	Moderate (ρ = 0.401; *p* = 0.001)	Volunteer 2 (ρ = 0.867; *p* = 0.012)
Mannitol	0–6 h	Non-significant (*p* = 0.118)	Non-significant (*p* > 0.05)
	0–15 h	Non-significant (*p* = 0.455)	Non-significant (*p* > 0.05)

* Correlations were calculated between gluten dose and the excretion of each parameter over two collection intervals (0–6 h and 0–15 h intervals). Correlation strength was categorized as weak (ρ < 0.4), moderate (0.4 ≤ ρ < 0.7), or strong (ρ ≥ 0.7). Results are shown both for all participants in each study and for the subgroup of 4 individuals who participated in all dosing sessions. Abbreviations: GIP, gluten immunogenic peptide; LMR, lactulose:mannitol ratio.

**Table 3:** CVs for GIP and LMR measurements by gluten dose and collection interval.

**u-GIP**	**CV (%)**	**CV (%)**
**Gluten Dose**	**0–6 h**	**0–15 h**
10 g	37.01	43.54
4 g	34.86	34.43
2 g	61.05	46.53
**LMR**	**CV (%)**	**CV (%)**
**Gluten Dose**	**0–6 h**	**0–15 h**
10 g	30.07	35.26
4 g	25.37	26.49
2 g	35.44	44.15

Abbreviations: GIP, gluten immunogenic peptide; LMR, lactulose:mannitol ratio; CV, coefficients of variation.

**Table 4:** Mean gluten antigen leakage index (GALI) across gluten doses and urine collection intervals.

Gluten Dose (g)	0–6 h GALI, Mean ± SD (µg/g)	0–15 h GALI, Mean ± SD (µg/g)	*n*
10	0.515 ± 0.178	0.966 ± 0.410	21
4	1.119 ± 0.416	1.733 ± 0.819	13
2	1.229 ± 0.750	1.933 ± 1.007	7

Abbreviations: GALI, gluten antigen leakage index; SD, standard deviation. *n*: number of unique volunteers who provided data for each specific dose–interval combination.

**Table 5:** Overview of study protocols and urine collection schemes included in the pooled analysis.

Protocol	Source	Gluten Doses (g)	Design	Urine Collection (Raw Sampling → Analysis Intervals)	Participants (*n*)
Study 1	Published [13]	10	Single intake	Void-by-void; pooled (0–6 h, 0–15 h)	15
Study 2	Published [14]	10	10 g repeated × 3 sessions (same participant)		12
New Protocol 1	New	0, 2, 4	3 sessions per participant (one per gluten dose: 0, 2, and 4 g)		7
New Protocol 2	New	4	Single intake	2 composites (0–6 h, 6–15 h)	12

Void-by-void indicates that each urination was collected separately and subsequently pooled to generate composite samples for the 0–6 h and 0–15 h intervals. “2 composites” indicates that urine was collected only as two composite fractions (0–6 h and 6–15 h), without separate void sampling.

## Data Availability

All data and analysis scripts used in this study are available from the authors upon reasonable request.

## Abbreviations

The following abbreviations are used in this manuscript:

GIP	Gluten immunogenic peptide
u-GIP	Urinary gluten immunogenic peptide
LMR	Lactulose:mannitol ratio
CD	Celiac disease
IQR	Interquartile range
CV	Coefficient of variation
GALI	Gluten antigen leakage index
